# A Scoping Review of Life-Course Psychosocial Stress and Kidney Function

**DOI:** 10.3390/children8090810

**Published:** 2021-09-16

**Authors:** Jesus Alejandro Estevez-Garcia, Marcela Tamayo-Ortiz, Alison P. Sanders

**Affiliations:** 1 Centre for Population Health Research, Environmental Health Department, National Institute of Public Health (INSP), Cuernavaca 62100, Mexico; jesus.estevez@espm.insp.mx; 2 Occupational Health Research Unit, Instituto Mexicano del Seguro Social, Mexico City 06720, Mexico; 3 Department of Environmental and Occupational Health, University of Pittsburgh, Pittsburgh, PA 15260, USA; APS109@pitt.edu

**Keywords:** psychosocial stress, early-life exposure, adverse life events, nephron endowment, kidney development

## Abstract

Increased exposure to maternal psychosocial stress during gestation and adverse neonatal environments has been linked to alterations in developmental programming and health consequences in offspring. A programmed low nephron endowment, among other altered pathways of susceptibility, likely increases the vulnerability to develop chronic kidney disease in later life. Our aim in this scoping review was to identify gaps in the literature by focusing on understanding the association between life-course exposure to psychosocial stress, and the risk of reduced kidney function. A systematic search in four databases (PubMed, ProQuest, Wed of Science, and Scopus) was performed, yielding 609 articles. Following abstract and full-text review, we identified 19 articles meeting our inclusion criteria, reporting associations between different psychosocial stressors and an increase in the prevalence of kidney disease or decline in kidney function, mainly in adulthood. There are a lack of studies that specifically evaluated the association between gestational exposure to psychosocial stress and measures of kidney function or disease in early life, despite the overall evidence consistent with the independent effects of prenatal stress on other perinatal and postnatal outcomes. Further research will establish epidemiological studies with clear and more comparable psychosocial stressors to solve this critical research gap.

## 1. Introduction

Kidney disease is a rapidly growing global health problem, both as a direct cause of morbidity and mortality as well as an important risk factor for cardiovascular disease [1,2]. The global all-age mortality rate from chronic kidney disease (CKD) increased 41.5% (95% Confidence Interval (CI): 35.2, 46.5) between 1990 and 2017. The global prevalence of CKD in 2017 was 9.1% (95% CI: 8.5, 9.8) after controlling for two primary comorbidities affecting kidney function: hypertension and diabetes. These trends indicate that CKD represents a serious threat to global public health and the economy [1,2]. Therefore, identifying preventable risk factors is essential for reducing CKD burden [3].

Stress-related psychosocial factors have repeatedly been associated with different non-communicable diseases, with studies linking stress to poorer kidney function [4,5], and prenatally, psychosocial stressors have been associated with adverse perinatal outcomes, including low birth weight, small for gestational age, and preterm birth [6,7]. However, the contribution of psychosocial stress to kidney development and diseases [8,9] has remained relatively unexplored.

The Developmental Origins of Health and Disease (DOHaD) hypothesis [10] postulates that adverse exposures during pregnancy or long before conception can result in poor fetal growth and impair normal development and organogenesis [6,11]. Nephrogenesis, the process of new nephron formation in the kidney, has been shown to be impacted by different harmful early-life exposures, including an inadequate diet, poor placental function, maternal stress, maternal smoking, and alcohol consumption, reducing the renal functional capacity (endowment) established at birth, which enhances an individual’s susceptibility to CKD and hypertension in later life [12,13,14]. There is an inverse correlation between the total number of nephrons and the risk of developing CKD and hypertension [13].

The purpose of this work is to provide an overview of the existing literature on exposure to psychosocial stress and effects on kidney function to advance conceptual clarity, synthesize the literature, and identify priorities for future research with a particular emphasis on early life exposures.

## 2. Materials and Methods

We followed the methodology by Arksey and O’Malley [15] and reported according to the guidelines for scoping reviews (PRISMA-ScR) [16].

The research question was: ”What is the contribution between exposure to psychosocial stress and kidney development and diseases?”. Search strategy results are defined in Table 1. The search was conducted during June 2021, spanning literature published between January 2000 and May 2021, using PubMed (MEDLINE), ProQuest Central, Web of Science, and Scopus. Manual searches of reference lists of systematic and other literature reviews identified additional primary studies. Literature was included if it primarily addressed (1) kidney function or diseases, (2) psychosocial stressors exposure in early or adult life (>18 years), (3) birth cohorts or longitudinal studies, or (4) experimental studies, in English.

We defined ‘psychosocial stressor’ as a life situation that creates an unusual or intense level of stress that may contribute to the development or aggravation of mental disorder, illness, or maladaptive behavior (i.e., divorce, the death of a child, prolonged illness, unwanted change of residence, a natural catastrophe, or a highly competitive work situation), in accordance with the American Psychological Association definition [17]. In addition, other psychosocial stressors were included according to the diagnostic and statistical manual of mental disorders DSM-IV-TR Axis IV in nine groups: problems with primary support groups, problems related to the social environment, educational problems, occupational problems, housing problems, economic problems, problems with access to health care, problems related to interactions with the legal system/crime legal charges, other psychosocial and environmental problems [18].

We excluded studies where the study population consisted of subjects with non-communicable disease (diabetes mellitus, hypertension, obesity or cancer) history. Additionally, conference abstracts, letter, notes, and comments were excluded.

**Figure 1:**
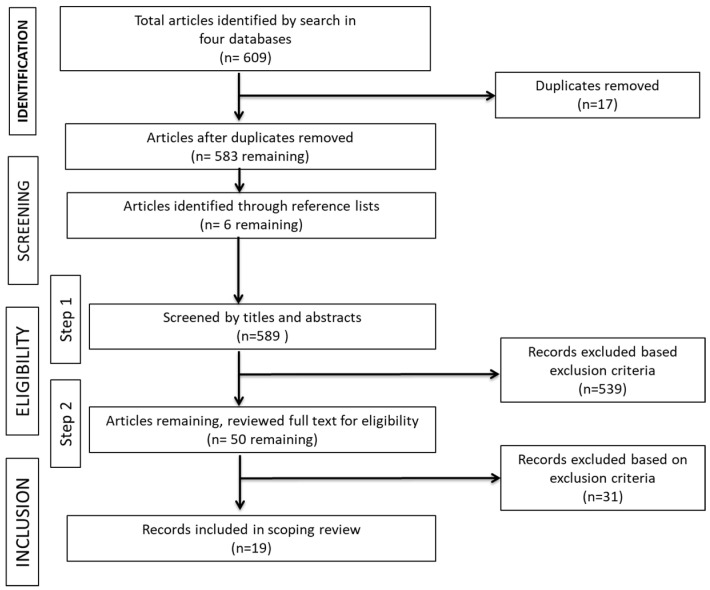
PRISMA flow diagram depicting the article selection process.

The PRISMA flow diagram depicting the article selection process is shown in Figure 1. Initial exclusions were made independently by J.A.E.-G and M.T.-O. based on title and abstract (Eligibility Step 1). J.A.E.-G reviewed full-text articles for eligibility and conferred with authors M.T.-O. and A.P.S. in cases of ambiguity (Eligibility Step 2).

Data were extracted using a predesigned form and included the year of publication, author, study design, country, population and settings, outcomes studied, primary results and conclusions.

## 3. Results

A total of 19 articles were identified an included in this review. Our main finding was the lack of human studies focusing on gestational exposure to psychosocial stress; this was mostly assessed in animal studies. The majority of epidemiologic studies examined adult psychosocial stressors (most often assessed by socio economic status (SES)) and reported inverse relationships with CKD status or kidney function, but this was not consistent across all studies: there were significant associations between low socioeconomic status with CKD, especially among women and African Americans [19,20,21,22,23]. Adjei et al. reported associations between stress at work/home and increased albuminuria and CKD risk [24]. Other psychosocial stress such as goal-striving stress (GSS) and stress-related disorders were associated with rapid kidney function decline (RKFD) after adjusting for demographics, health behaviors, risk factors, and the burden of discrimination [3,25,26]. In contrast, higher optimism was associated with lower odds of CKD and lower odds of RKFD [27].

### 3.1. Study Designs and General Characteristics

Articles included *n* = 2 cross-sectional studies [19,24], *n* = 1 retrospective cohort [20], *n* = 8 prospective cohort studies [3,19,21,22,23,25,26,27,28] and *n* = 8 animal model studies [29,30,31,32,33,34,35,36]. The epidemiological studies included data for adults with different age distributions, ethnic compositions and who lived in both urban and rural areas. Seven of the studies were carried out in the United States (Jackson Heart Study, Healthy Aging in Neighborhoods of Diversity across the Life Span HANDLS study, Life Course Socioeconomic Status Study), one in Korea (KoGES study), Sweden (SCREAM project), and Ghana–Netherlands–Germany–United Kingdom (Research on Obesity & Diabetes among African Migrants RODAM study). Table 2 summarizes the results of these studies, starting with epidemiological studies followed by experimental studies.

### 3.2. Psychosocial Stressors

The included epidemiological studies used a variety of methodologies to define and assess psychosocial stress as different explanatory variables: global perceived stress [33], low socioeconomic status [19,20,22,23], perceived discrimination [28], four constructs of psychological stress [24], psychosocial well-being [3], dispositional optimism [27], goal-striving stress [26], and clinical diagnosis of stress-related disorders [25]. The authors describe the use of self-administered questionnaires, including specific questions within a validated tool (e.g., Psychosocial Well-being Index short-form [3], 6-item Life Orientation Test-Revised scale LOT-R, INTERHEAR´s psychological stress scale, list of threatening experience—LTE, Patient Health Questionnaire—PHQ 9, 9-item Discrimination Scale of the Experience of Discrimination questionnaire, Socioeconomic Status) [3,19,20,21,22,24,27,28].

The experimental studies included rodent (rat and mouse) species. Researchers used behavioral stress models where the pups were exposed to early-life stress (mainly postnatal period) by immobilization [31,32], maternal separation (MatSep) [29,36], glucocorticoids infusion [34,35] and transportation protocols [36].

### 3.3. Kidney Outcomes

Kidney function among population-based studies included baseline and repeated measures of serum/plasma creatinine and urinary albumin. The primary outcomes were the prevalence or incidence of CKD and CKD progression assessed through the estimated glomerular filtration rate (eGFR) decline [3,26,27]. GFR was estimated for using either the “Modification of Diet in Renal Disease (MDRD) Study” [19,20,22] or the Chronic Kidney Disease Epidemiology Collaboration equation models [3,24,25,26,27,37].

In animal studies, a variety of post-mortem renal histopathology analyses (estimation of the total number of glomeruli per kidney by stereological counting [31,32,34,35] and α-adrenergic receptors α-ARs), plasmatic and urinary creatinine samples [37], renal inflammatory and metabolic biomarkers [30,36] were carried out in exposed and control groups to examine stress-induced effects. Prenatal exposure to glucocorticoids resulted in postnatal growth restriction [34], with significant reduction in the kidney weight and total nephron number, increased mean arterial pressure, presence of albuminuria, and the expression of receptors in the renin–angiotensin system and apoptotic gene markers in offspring [34,35].

The experimental studies in postnatal exposure (prepuberal age) to physical immobilization, maternal separation, or transportation stress models showed significantly lower values of kidney weight, and glomerular volume density in the stressed group [31,32], as well as sensitization to arterial hypertension after angiotensin II infusion [33], exaggerated gene expression immune response (IL-1β and T cells) [36], reduced alfa-adrenergic receptor (α-AR) density in renal vasculature [29], and cardiorenal metabolic alterations [30].

## 4. Discussion

The literature search for this review focused on exposure to psychosocial stress and kidney function, and consistent with prior reviews [8,25], we note that a growing body of evidence supports a link between lifetime stressors and CKD risk or kidney function decline. However, we note that there is an important gap in research for studies that examined early-life or prenatal stress. Only two animal studies and one epidemiological study indirectly addressed this research question [33,36,38]. Specifically, we found no studies addressing psychosocial determinants of kidney health in early childhood, an area that remains a critical research need [39]. We included studies that reported associations between different psychosocial stressors (low socio-economic status, racial discrimination, migration, etc.) and poorer kidney health outcomes in previously healthy adult populations. 

Individuals can be exposed to psychosocial stressors through different pathways, including poverty, low socioeconomic status, life events, pregnancy-related stressors, racial discrimination, and the presence of stress factors related to living conditions and the geographic area. These psychosocial stress factors have previously been associated with adverse perinatal effects that include low birth weight, small for gestational age, prematurity, neurodevelopment, metabolic, cardiovascular, and respiratory alterations [6,7,40,41,42,43]. Additionally, it is essential to acknowledge that exposure to chemical substances may have a synergistic effect in perinatal outcomes when pregnant women are co-exposed to psychosocial stressors and other environmental toxicants, both individually and at a community level [44,45,46]. As is the case for preterm birth, wherein concomitant exposure to adverse life events (e.g., poverty, racism, inequitable access to healthcare) as well as an individual’s exposure to environmental toxicants (e.g., air pollution, metals, some pesticides, or phthalates), may also damage developing kidneys, reducing nephron endowment over the life course [38,47,48]. Co-exposures, or studies where psychosocial stress may be the result of other life events such as starvation, were outside the scope of this review.

Different biological mechanisms that enhance disease and frailty in adulthood have been described to explain early-life exposure to biologic and psychosocial stressors [49]. Specifically, regarding kidney development, nephrogenesis culmination between gestational weeks 34 and 36, together with nephron maturation in the early postnatal period, are critical windows of susceptibility [9]. Several studies demonstrated that low birth weight (<2500 g), prematurity and delayed intrauterine growth are associated with CKD in adulthood, secondary to elevated blood pressure, microalbuminuria presence and reduced estimate glomerular filtration rate [50,51], because low birth weight correlates linearly with nephron number in children and adults [12,52].

From a neuroendocrine and epigenetics perspective, there is evidence suggesting that fetal, placental and maternal factors, associated through the multiple-hit hypothesis, influence the development of antenatal distress and adverse offspring outcomes by different pathways [12,53]. An adverse environment induces the long-lasting hyper-reactivity of physiological survival systems: stress responses on the maternal and fetal hypothalamic–pituitary–adrenal (HPA) axis [54,55,56], immune and inflammatory responses [57,58], and energy-conserving responses [59]. Particularly, exposure to maternal–fetal undernutrition or psychosocial stress for a certain organ can impair growth, resulting in a permanently reduced number of normally functioning units (e.g., nephrons, cardiomyocytes, pancreatic insulin-secreting Β cells) [60]. At the same time, reduced functional capacity can limit the ability of the organ to adapt to an increased functional load imposed postnatally, and increase the risk for later life kidney disease, heart failure, and diabetes [61]. Specifically, current evidence shows that the developmental programming of chronic conditions, such as diabetes, cardiovascular disease, obesity, and depression, has a transgenerational effect [11,62] through the excessive expression of the glucocorticoid-responsive transcription factor called nuclear-receptor related 1 protein (NURR1) with environmental stress interactions during early life [63].

Regarding the developmental programming field, human studies are limited due to challenges in the inherent temporal lag between an adverse event during pregnancy and the occurrence of an associated clinical phenotype. Therefore, animal models are critically important in exploring possible mechanisms for kidney development under controlled conditions and in discrete developmental periods [64]. The correct assessment of the timing of psychosocial stress exposure in pregnancy may be key to determining potential fetal programming effects [64], because in humans and species such as sheep, nephrogenesis is largely completed before birth, whereas in rodents, rabbits, and pigs, nephron formation and completion continue in the postnatal period [65,66]. Furthermore, many psychosocial stressors cannot be recreated or simulated in a laboratory setting.

The results of animal studies subject to different stress models report that prenatal and postnatal exposure to glucocorticoids is associated with: a reduced renal reserve [31,32,34,35,67,68], changes in the vascular response to vasoconstrictors [29,69], activity increase in the renin–angiotensin–aldosterone system and its renal and brain receptors [31,35], change in molecular pathways that regulate the kidney transcriptome [36,70], alterations of the immuno-inflammatory processes [71] and modifications in the baroreceptors response [29,35,72]; each of these factors jointly contributes to altered kidney development and the potentially increased risk of later life arterial hypertension and CKD [34,53,73]. Additionally, recent evidence suggests a transgenerational transmission of these alterations through epigenetic mechanisms such as chromatin remodeling and modifications of non-codifying RNA [11,52].

During pregnancy, psychosocial stressors may contribute to the persistence of disparities of adverse birth outcomes across specific socioeconomic and ethnic groups. Higher levels of psychosocial stress may be associated with lower SES populations, where food insecurity, greater financial strain, and job strain are more prevalent compared to higher SES groups [74]. Perceived stress and depression may also be higher among non-white women as a result of racial discrimination [28]. The Cumulative Disadvantage Theory highlights how early social and economic advantage or disadvantage shapes the health outcomes of socially defined groups over time [8]. However, Luneyra et al. concluded that life stressors were negatively associated with prevalent CKD at baseline in the Jackson Heart Study cohort. It is important to understand the underlying factors driving the unexplained disparities in CKD outcomes among African Americans; therefore, future studies should delineate the potential role of biological markers in the relationship between psychosocial factors and kidney health [33], while recognizing genetic factors associated with CKD in risk groups such as African Americans.

This review has some limitations. The search strategy used terms that included different psychosocial stress factors. However, it is not an exhaustive list that includes all possibilities and interactions with others biological, genetics, quality of healthcare systems, maternal comorbidities, family support, and environmental factors to address their cumulative effects in the nephron endowment. We only included studies published in English between 2007 and 2020, carried out in high-income countries. Due to the heterogeneity of articles identified, we could not find a definitive answer to our research question. We did not formally evaluate the level of evidence of the studies because this was not the main goal of our scoping review. It is also important to highlight that some of the epidemiological evidence arose from a single cohort study (the Jackson Heart Study), although applied different metrics for the evaluation of psychosocial stressors. Our findings provide a guide for future prospective epidemiological studies or controlled experimental trials that interrogate the causal roles of psychosocial early-life stress and kidney function, considering the timing of outcome and exposure in the critical windows of susceptibility, and other risk factors such as the dehydration and sex effects [3,12,25].

Finally, findings between studies can also vary considerably depending on the methodological approaches to measuring psychosocial stress. Self-report questionnaires may be biased due to either over- or under-reporting. By measuring clinical parameters, e.g., cortisol levels, blood pressure or heart rate, it is possible to better evaluate the response to stress [75,76], although not always feasible.

## 5. Conclusions

Epidemiological evidence in adult populations shows the association of different psychosocial stressors on kidney function, and the overall evidence in animal studies is consistent with the independent effects of prenatal psychosocial stress on perinatal and postnatal outcomes. We conclude that there is an important gap to fill with human studies between exposure to psychosocial stress during pregnancy and kidney outcomes.

## Figures and Tables

**Table 1:** Search strategy, terms, and results for traditional databases.

Step	Search Description	Results			
		PUBMED	SCOPUS	ProQuest	Web of Science
1	“stress, psychological” [MeSH Terms] OR “stress disorder *” [All Fields] OR “stress disorders, traumatic” [MeSH Terms] OR “psychosocial stress *” [All Fields] OR “maternal depressi *” [All Fields] OR “Adjustment Disorders” [MeSH Terms] OR “Anxiety Disorders” [MeSH Terms] OR “adverse life event *” [All Fields] OR “Early life stress” [All Fields] OR “Intrauterine stress” [All Fields] OR “prenatal stress *” [All Fields] OR “maternal stress” [All Fields] OR “Intimate Partner Violence” [MeSH Terms]	282,346	305,521	637,299	384,716
2	1 AND “kidney function tests” [MeSH Terms] OR “kidney function” [Text Word] OR “renal function” [Text Word] OR “Acute Kidney Injury” [MeSH Terms] OR “kidney failure, chronic” [MeSH Terms] OR “Glomerular Filtration Rate” [MeSH Terms] OR “renal function decline” [Text Word]	522	723	3895	3900
3	2 AND (“birth cohort*”[Text Word] OR “Longitudinal Studies”[MeSH Terms] OR “Cohort Studies”[MeSH Terms])	57	44	209	299
Total		609			
Excluding duplicates		26			

Dates included in search: 1 January 2000–2021. Searches were conducted on 30 May 2021.

**Table 2:** Findings from the included articles reporting the association between psychosocial stressors and kidney outcomes in experimental and epidemiological studies.

Epidemiological Studies			
Population	Exposure	Outcome	Key Findings
**1. Shoham et al., 2007**			
*n* = 12,631 adults Life Course Socioeconomic Status (LCSES) ancillary study retrospective cohort.	Social class, education level, or area-level socioeconomic resources in childhood and adulthood	Increased risk of adult kidney disease.	Socioeconomic factors, including area socioeconomic status and social class were associated with CKD and may account for some observed racial disparities
**2. Bruce et al., 2010**			
*n* = 5301 Jackson Heart Study (JHS) prospective cohort.	Socioeconomic status (SES)	Chronic kidney disease (CKD)	High SES was associated with lower risks for CKD, although the results were not linear.
**3. Crews et al., 2010**			
*n* = 2375 Healthy Aging in Neighborhoods of Diversity Across the Lifespan (HANDLS) Study	Individual-level SES, race	Chronic kidney disease (CKD)	Individual-level poverty (low SES) was associated with prevalent CKD among African Americans, but not among whites Among African Americans, low SES was independently associated with a nearly twofold greater risk of CKD when compared with higher SES
**4. Beydoun et al., 2017**			
*n* = 1620 HANDLS	Self-reported perceived racial discrimination, perceived gender discrimination	Chronic kidney disease (CKD)	Poor kidney function assessed by glomerular filtration rate The strength of associations differed by sex and race groups
**5. Lunyera et al., 2018**			
*n* = 5301 JHS	Non-depressive psychosocial factors	Chronic kidney disease (CKD)	Life stressors were inversely associated with prevalent CKD at baseline. No other associations between psychosocial factors and CKD outcomes were evident after a median follow-up of 8 years.
**6. Adjei DN et al., 2019**			
*n* = 5659 Research on Obesity & Diabetes among African Migrants (RODAM) multicenter cross-sectional study	Psychosocial stressors (discrimination, perceived stress at work or at home, negative life events and depressive symptoms)	Chronic kidney disease (CKD)	Positive association between stress at work/home and albuminuria and CKD risk.
**7. Cain-Shields et al., 2020**			
*n* = 5301 JHS	Goal-striving stress	Rapid kidney function decline	Stress related to not achieving goals was associated with a greater risk of rapid kidney function decline
**8. Glover LM et al., 2020**			
*n* = 5301 JHS	Dispositional optimism	Chronic kidney disease (CKD) Rapid kidney function decline	Higher optimism was associated with lower odds of CKD and lower odds of rapid kidney function decline
**9. Kim JY. et al., 2020**			
*n* = 7246 Korean Genome and Epidemiology Study (KoGES)	Psychosocial distress	Annual glomerular filtration rate (eGFR) decline	Higher levels of psychosocial distress were closely associated with an increased risk of rapid kidney function decline Increase in risk was independent of sociodemographic characteristics or behavioral patterns
**10. Lunyera et al., 2020**			
*n* = 5301 JHS	Cumulative lifetime socioeconomic status (SES), allostatic load mediation	Chronic kidney disease (CKD)	Lower cumulative lifetime SES associated with baseline CKD prevalence directly and indirectly via allostatic load Modestly associated with CKD incidence and eGFR decline via baseline allostatic load
**11. Su G et al., 2021**			
*n* = 30,998 Stockholm CREAtinine Measurements-SCREAM project	Stress-related disorders (SRD)	Risk of chronic kidney disease (CKD) progression Acute kidney injury (AKI)	SRDs were associated with a subsequent risk of AKI and CKD progression, independent of history of other psychiatric disorders, comorbidities and medications Risk of AKI highest within the first year from SRD diagnosis, CKD risk sustained over time
**Experimental Studies**			
**Population**	**Exposure**	**Outcome**	**Key findings**
**1. Singh et al., 2007**			
*n* = 20 Sprague Dawley rats	- Prenatal - Maternal natural glucocorticoid corticosterone (CORT) treatment	Renin–angiotensin system (RAS) of the embryo and adolescent offspring.	Nephron deficit and development of hypertension in rat offspring. Outcomes not influenced by birth weight In the embryonic period, altered expression of receptors of RAS may have contributed to nephron deficit In the postnatal period, it may have contributed to hypertension Study suggests that increased physiological levels of CORT can program similar changes to those seen with pharmacological doses of the synthetic glucocorticoid
**2. DeSouza et al., 2011**			
*n* = 15 Wistar male rats 4-week-old (prepuberal)	- Physical immobilization until 9 weeks of age (*n* = 8) - Control group (*n* = 7)	Kidney morphometrical analysis Total number of glomeruli per kidney and glomerular volume density. Adrenal mass index, creatinine, and testosterone serum concentration	Chronic stress before puberty causes an important reduction in the number of nephrons (36%) in rats without raising serum creatinine Morphological alterations may have serious implications predisposing individuals to renal disease and hypertension in adult life
**3. Loria et al., 2013**			
*n* = 6–8/group Wistar Kyoto rats	Maternal separation (MatSep) f postnatal period (days 2 to 14)	Mean arterial pressure (MAP) and heart rate (HR)	The maternal separation stress model, MatSep, sensitizes chronic blood pressure responses to angiotensin II in renal phenotype in male but not female rats
**4. O’Sullivan et al., 2015**			
*n* = 64 Pregnant mice	Prenatal corticosterone (CORT) *n* = 32 Control *n* = 32	- Blood pressure - Renal gene expression - Nephron endowment - Responses to increased dietary sodium	Prenatal CORT exposure reduced nephron endowment and caused albuminuria in male and female offspring; however, CORT-exposed male offspring were hypotensive in a salt-loading challenge, indicating that renal changes (that are normally associated with increased blood pressure) were being overridden by other factors
**5. Loria et al., 2017**			
*n* = 9 Adult male Wistar Kyoto rats	Maternal separation (MatSep, *n* = 5) stress model from postnatal period (days 2 to 14) Control group (*n* = 4)	Renal α-adrenergic receptor density Renal vascular responsiveness to adrenergic stimulation	MatSep-induced overactivation of the renal neuroadrenergic tone (1) Lower density of α-ARs in renal vasculature; (2) Attenuated renal vasoconstrictor responses to acute α-adrenergic stimulation; (3) Diminished baroreceptor control of heart rate.
**6. Marchon et al., 2018**			
*n* = 76 Wistar rats Prepubertal (4 weeks old) *n* = 40 Adult rats (10 weeks old) *n* = 36	Immobilization	Renal morphological alterations	Irreversible glomerular loss Renal impairment was interrupted by removal of the stress stimuli
**7. De Miguel et al., 2018**			
*n* = 4–9/group Wistar Kyoto rats Adult male (12 weeks old)	Maternal separation (MatSep)	Renal inflammatory state	“Priming” or sensitization of the immune system, resulting in an exaggerated gene expression response to an immune challenge in adulthood Early-life stress-model-mediated sensitization of the immune system may play an important role in promoting cardiovascular disease earlier and more robustly in adulthood
**8. Poplawski et al., 2020**			
*n* = 23 C57BL/6 Mus musculus mice, adult male stress animals *n* = 14, control *n* = 9	Early-life transportation stress model was applied in mothers and their pups	Cardiorenal metabolism	Altered (i) organ weights, (ii) affective state, and (iii) metabolites and/or metabolic pathways linked to adverse mental health outcomes and metabolic illness, such as cardiorenal syndrome, insulin resistance, diabetes, and obesity Stress-associated metabolic signatures in somatic organs may provide early predictors of health risks in later life and reveal new candidates for peripheral biomarker detection with diagnostic value

## Data Availability

Not applicable.
